# Association of intrinsic capacity with functional ability, sarcopenia and systemic inflammation in pre-frail older adults

**DOI:** 10.3389/fmed.2024.1374197

**Published:** 2024-03-06

**Authors:** Reshma Aziz Merchant, Yiong Huak Chan, Denishkrshna Anbarasan, Bruno Vellas

**Affiliations:** ^1^Division of Geriatric Medicine, Department of Medicine, National University Hospital, Singapore, Singapore; ^2^Department of Medicine, Yong Loo Lin School of Medicine, National University of Singapore, Singapore, Singapore; ^3^Biostatistics Unit, Yong Loo Lin School of Medicine, National University of Singapore, Singapore, Singapore; ^4^Gérontopôle, Centre Hospitalier Universitaire de Toulouse, Toulouse, France

**Keywords:** intrinsic capacity, sarcopenia, systemic inflammation, pre-frail older adults, growth differentiation factor 15

## Abstract

**Background:**

Decline in intrinsic capacity (IC) has been shown to accelerate progression to disability. The study aims to explore association of IC composite score with functional ability, sarcopenia and systemic inflammation in pre-frail older adults.

**Methods:**

Cross-sectional study of pre-frail older adults ≥60 years old recruited from the community and primary care centers. Composite scores of four domains of IC were measured: locomotion, vitality, cognition and psychological. FRAIL scale was used to define pre-frailty. Muscle mass was measured using the bioelectrical impedance analysis. Systemic inflammation biomarkers [Interleukin-6 (IL-6), Interleukin-10 (IL-10), Tumor Necrosis Factor Alpha (TNF-α), and Growth differentiated factor 15 (GDF-15)] were measured. Participants in the lowest tertile (T1) exhibited greater decline in IC.

**Results:**

A total of 398 pre-frail older adults were recruited, mean age was 72.7 ± 5.8 years, 60.1% female, education level 7.8 years, and 85.2% were of Chinese ethnicity. A total of 75.1% had decline in locomotion, 40.5% in vitality, 53.2% in cognition and 41.7% in psychological domain. A total of 95% had decline in at least one domain. T1 was significantly associated with ADL impairment (aOR 3.36, 95% CI 1.78–6.32), IADL impairment (aOR 2.37, 95% CI 1.36–4.13), poor perceived health (aOR 0.96, 95% CI 0.95–0.98), fall (aOR 1.63, 95% CI 1.05–2.84), cognitive impairment (aOR 8.21, 95% CI 4.69–14.39), depression (aOR 101.82, 95% CI 33.62–308.37), and sarcopenia (aOR 2.40, 95% CI 1.60–5.45). T1 had significant associations with GDF-15, IL-10, and IL-10 to TNF-α ratio.

**Conclusion:**

Decline in IC composite score among pre-frail older adults was associated with functional limitation, sarcopenia, and systemic inflammation.

## Introduction

Population aging is a global phenomenon and by 2050, the number of older adults aged 65 years old and above will double to 1.5 billion (1). Aging is associated with decline in hearing, vision, mobility, and cognition, along with an increased prevalence of non-communicable diseases. These factors collectively contribute to the risk of frailty, dementia, disability, and mortality (2). Countries worldwide, including Singapore are adopting a population-wide approach to healthy aging prioritizing preventive health measures to mitigate long term health and social care cost (3). Functional ability encompasses both physical and cognitive ability, and is determined by the interaction of intrinsic capacity (IC) with environment (4). IC was first described in the World Report on Ageing and Health as a composite of physical, cognitive, psychological, vitality and sensory capacities. It is a paradigm shift from the usual disease-based approach to function and physiological reserve concept which has a better predictive ability for functional decline and incident disability (5, 6). The decline in IC can lead to increased socio-economic cost, participation restriction, frailty, disability, social isolation, and mortality (7, 8). In 2019, the World Health Organization published The Integrated Care for Older People (ICOPE) care pathway. This approach recommends screening for IC decline followed by person-centered assessment, personalized intervention, and monitoring plan (1, 4).

Frailty is a multi-dimensional dynamic construct caused by decline in physiological reserve which increases vulnerability to adverse outcomes when exposed to stressors (9). IC serves as an indirect measure of physiological reserve. Sarcopenia, defined as age related decline in muscle mass accompanied by reduced muscle strength, or physical performance is a component of physical frailty (10). It is increasingly recognized as a global health problem due to its association with morbidity, mortality and many chronic diseases such as fatty liver disease, dementia and diabetes mellitus (10–12). Notably, the co-existance of frailty with conditions such as liver fibrosis is associated twice the risk of overall mortality (13). Frailty, sarcopenia and IC decline can co-exist in the same individual accelerating the onset of disability (8, 14). Pre-frailty is a transition phase from robust to frailty with a prevalence rate ranging from 34.6 to 50.9% depending on the population studied and the frailty screening tools used (15). Liu et al demonstrated that 83.3% of pre-frail older adult have at least one IC impairment at baseline (16). Impairment in any of the IC domains in pre-frail older adults have shown to accelerate frailty progression (17–19).

Systemic inflammation is a well-known hallmark of aging and is associated with dementia, frailty, and sarcopenia (20). A systematic review reported significant association of plasma c-reactive protein (CRP), interleukin-6 (IL-6), and tumor necrosis factor receptor-1 (TNFR-1) with frailty (20). In addition, Lu et al. (21) recently showed that lower baseline IC was associated with higher inflammatory biomarkers such as plasma CRP, IL-6, TNFR-1, and growth differentiation factor-15 (GDF-15) (21). GDF-15 is also known as macrophage inhibitory cytokine-1 and recognized as a biomarker for mitochondrial dysfunction. It is both a pro-inflammatory and anti-inflammatory cytokine where it exerts protective effect through immune-modulatory function and serves as a poor prognostic biomarker in myopathies, cancer, and cardiovascular disease (22). IL-10 is a potent anti-inflammatory cytokine and increases in response to inflammatory cascade (23). Besides its role in neurodegenerative diseases, low or absent IL-10 in mice has shown to be associated with frailty (24, 25).

While the concept and definition of IC has been accepted by most researchers globally, the measurement of IC remains an area of ongoing debate. There is yet no ideal measurement tools nor calculation models for IC composite scores. There is a suggestion that IC should be interpreted as a composite score and a system as the outcomes are determined by dynamical interrelations between domains which share common biological pathways (26). To date, there are limited studies on association of IC composite scores with functional ability, systemic inflammation, and sarcopenia in pre-frail older adults who are at highest risk of progressing to frailty and disability. There is only one recent study which reported association of IC with sarcopenia in hospitalized older person (14). The aim of this study is to explore association of IC composite score with functional ability, sarcopenia and systemic inflammation in pre-frail older adults.

## Materials and methods

### Study participants

This is a secondary cross-sectional analysis of baseline data from a multidomain intervention study in pre-frail older adults ≥60 years old recruited from the community and primary care centers in Singapore between February 2019 and May 2022 (27, 28). A total of 502 participants were recruited but complete demographic information and body composition analysis were available for 398 participants due to constraints of COVID-19 restrictions. Participants were screened for frailty using the FRAIL scale and pre-frailty was defined by a score of 1–2 out of a maximum score of 5 (29). The details on recruitment, biomarkers and interventions are described in earlier studies (27, 28). Recruited participants should be able to provide consent, follow instructions and ambulant. Exclusion criteria included nursing home residents, presence of pacemaker or defibrillator and underlying psychiatric conditions. This study conformed to the principles of the Declaration of Helsinki and was approved by The National Healthcare Group Domain Specific Review Board (Reference: 2018/01183 and 2019/00017). Informed consent was obtained from all participants involved in the study.

### Intrinsic capacity

Four domains of intrinsic capacity (IC) were evaluated–locomotion, vitality, cognition and psychological (Supplementary Table 1). Participants were given a score from 0 to 2 for each domain. Score of 0 or 1 indicates a decline and 2 no decline, with a total score of 8 (lower scores representing greater IC decline) (30). Locomotion was assessed using four meter gait speed and 5x sit-to-stand (5x STS) timing. The 5x STS timing was measured by the time taken to stand five times consecutively from a seated position without any arm support. Gait speed <1 m/s and/or 5x STS < 12 s were considered impaired locomotion (31). Vitality domain comprised of nutritional status and appendicular skeletal muscle index (ASMI). The Mini Nutritional Assessment-Short Form was used to evaluate nutritional status. With a maximum score of 14, <8 indicates malnourished and 8–11 at risk of malnutrition (32). Body composition was assessed using the InBody S10 multi-frequency bioelectrical impedance analyzer. InBody S10 provides results on segmental lean analysis and ASMI was calculated based on sum of lean mass of 4 limbs divided by height squared. Low ASMI was defined as <7.0 kg/m^2^ for males and <5.7 kg/m^2^ for females based on the Asia Working Group for Sarcopenia (AWGS) 2019 consensus criteria (31).

Cognition domain was evaluated using the Montreal Cognitive Assessment (MoCA) and self-reported subjective cognitive decline. With a maximum score of 30, participants scoring <26 were considered cognitively impaired (33). Subjective cognitive decline (SCD) was defined based on a question “do you feel that you have more problems with memory than most?” (34). Psychological domain was evaluated using the 15-item Geriatric Depression Scale (GDS-15), and a single question from the EuroQol-5 Dimensions (EQ-5D) question on anxiety/depression. Depression was defined as GDS-15 score >5 (35). The EQ-5D question scoring ranged from 0 (not anxious/depressed), 1 (slight anxious/depressed) to 4 (extremely anxious/depressed) (34, 36). Scoring and distribution are summarized in Supplementary Table 1. Participants were then split into tertiles based on their total IC score.

### Co-variates

Trained research assistants administered the study protocol gathering information on demographics, medications, chronic diseases, cognition, falls, sarcopenia, functional status, pain, and perceived health. Polypharmacy was defined as taking ≥5 medications daily and multimorbidity as ≥2 chronic diseases (hypertension, hyperlipidaemia, diabetes, heart disease, stroke, cancer, peripheral arterial disease, lung disease, kidney disease, osteoporosis). Perceived health was assessed using the EuroQoL Visual Analog Scale (36). Activities of daily living (ADL) was evaluated using Katz’s ADL questionnaire with a maximum score of 6 and instrumental activities of daily living (IADL) using the Lawton and Brody’s IADL questionnaire with a maximum score of 8 (37, 38). The Rapid Physical Assessment (RAPA) was used to assess physical activity (39).

Maximum handgrip strength (HGS) was measured in a seated position using the Jamar hand dynamometer on with elbow flexed at 90°. Low HGS was defined as <28 kg for males and <18 kg for females (31). The Short Physical Performance Battery (SPPB)–scored with a maximum of 12 points across three components–balance, gait and chair-stand was also administered. SPPB < 9 was considered poor performance (31). Four-meters gait speed was measured with 3 m of acceleration and deceleration path.

### Muscle mass indices and sarcopenia

Readings for body cell mass (BCM), and appendicular skeletal mass (ASM) were obtained from InBody S10 multi-frequency bioelectrical impedance analyzer. Diagnosis of sarcopenia was based on the 2019 Asian Workgroup for Sarcopenia (AWGS) criteria (31).

### Inflammatory biomarkers

GDF-15, IL-6, IL-10 and Tumor Necrosis Factor-Alpha (TNF-α) cytokines were measured by accredited hospital-based laboratory. Enzyme-linked immunosorbent Assay was used to measure GDF-15 with a detection range of 2.0–2400 pg./mL and IL-10 with a detection range of 2.0–400.0 pg./mL. IL-6 was measured using the electrochemiluminescence immunoassay (ECLIA) with a detection range between 1.5 and 50 000 pg./mL. Immunoenzymetric assay was used to measure TNF-α cytokine with a detection range between 1.0 and 498 pg/mL. The ratio of IL-10 to TNF-α was also calculated.

### Statistical analysis

All analyses were conducted using SPSS 28.0. Statistical significance was set at a two-sided 5% level. Descriptive analyses for categorical and continuous variables were presented as frequencies with percentages and mean ± standard deviation, respectively. Univariate analysis for numerical measures across the groups was performed using the Welch test to account for unequal sample sizes, followed by Games-Howell *post-hoc* tests for pairwise comparisons. For categorical variables, we used Chi-Square and Fisher’s Exact Test, with Bonferroni’s correction. Baseline plasma biomarker levels were summarized as medians with interquartile range. We compared baseline values using Mood’s median test.

Multinomial regression was conducted to investigate the association between IC and body composition, comparing it to participants with better IC in Tertile 3 (T3). We adjusted for age, gender, ethnicity, education years, and physical activity. Both unadjusted and adjusted odds ratios (ORs), along with their 95% confidence intervals (CIs), were reported. Additionally, we employed quantile regression to explore the relationship between IC and plasma biomarkers. Again, we provided unadjusted and adjusted β-coefficients, along with their 95% CIs.

## Results

### Participant characteristics and demographics

**FIGURE 1:**
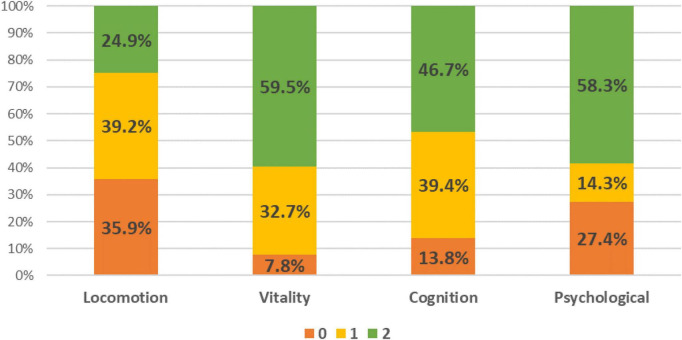
Distribution of individual intrinsic capacity domain score.

The decline in individual IC domain was 75.1% in locomotion, 40.5% in vitality, 53.2% in cognition and 41.7% in psychological domain (Figure 1). Amongst them, 95.0% had decline in at least one domain, 68.6% in two and 34.5% in three and 12.6% in all four domains (Supplementary Table 2). Of the 398 older adults, mean age was 72.7 ± 5.8 years, 60.1% female, education level 7.8 ± 4.4 years, and 85.2% were of Chinese ethnicity (Table 1). Amongst them, 34.2% were in Tertile 1 (T1), 22.1% in Tertile 2 (T2), and 43.7% in T3. Those in T3 were the youngest (71.0 ± 5.2 years), followed by T2 (73.0 ± 6.1 years), and T1 (74.3 ± 6.1 years). Participants in T1 had the lowest education level (6.4 ± 4.2 years) vs. T2 (8.1 ± 4.2 years) and T3 (8.8 ± 4.4 years). Perceived health rating was significantly lower in T1 (65.5 ± 15.6) compared with T3 (72.1 ± 13.8).

**TABLE 1:** Baseline characteristics.

	Overall *n* = 398	Tertile 1 *n* = 136 (34.2%)	Tertile 2 *n* = 88 (22.1%)	Tertile 3 *n* = 174 (43.7%)	*P*-value
**Demographics**					
Age, years (range)	72.70 (60–91)	74.26 (63–91)	72.95 (61–91)	71.36 9 (60–85)	**<0.001**
Gender					0.076
Male	159 (39.9)	44 (32.4)	37 (42.0)	78 (44.8)	
Female	239 (60.1)	92 (67.6)	51 (58.0)	96 (55.2)	
Ethnicity					0.737
Chinese	339 (85.2)	113 (83.1)	75 (85.2)	151 (86.8)	
Malay	25 (6.3)	11 (8.1)	7 (8.0)	7 (4.0)	
Indian	31 (7.8)	11 (8.1)	5 (5.7)	15 (8.6)	
Others	3 (0.8)	1 (0.7)	1 (1.1)	1 (0.6)	
BMI, kg/m^2^	25.44 ± 4.64	24.80 ± 5.21	25.47 ± 4.71	25.92 ± 4.08	0.106
Education, years	7.83 ± 4.38	6.44 ± 4.15	8.11 ± 4.22	8.76 ± 4.39	**<0.001**
**Chronic disease**					
Hypertension	270 (67.8)	94 (69.1)	59 (67.0)	117 (67.2)	0.834
Hyperlipidaemia	300 (75.4)	102 (75.0)	68 (77.3)	130 (74.7)	0.901
Diabetes	184 (46.2)	67 (49.3)	37 (42.0)	80 (46.0)	0.569
Multi-morbidity	307 (77.1)	107 (78.7)	68 (77.3)	132 (75.9)	0.842
Polypharmacy	112 (28.1)	38 (27.9)	23 (26.1)	51 (29.3)	0.865
Perceived health rating	69.39 ± 14.34	65.50 ± 15.55	69.73 ± 12.23	72.14 ± 13.77	**<0.001**
RAPA total	3.25 ± 1.56	2.92 ± 1.43	3.22 ± 1.61	3.52 ± 1.59	**0.003**
MoCA total	25.28 ± 4.41	22.79 ± 5.22	25.61 ± 4.04	27.06 ± 2.63	**<0.001**
≥1 Fall in 12 months	103 (25.9)	46 (33.8)	18 (20.5)	39 (22.4)	**0.032**
Depression	109 (27.4)	86 (63.2)	19 (21.6)	4 (2.3)	**<0.001**
Subjective cognitive decline	106 (26.6)	60 (44.1)	26 (29.5)	20 (11.5)	**<0.001**
≥1 ADL impairment	79 (19.8)	42 (30.9)	18 (20.5)	19 (10.9)	**<0.001**
≥1 IADL impairment	109 (27.4)	57 (41.9)	19 (21.6)	33 (19.0)	**< 0.001**
Nutrition status					**<0.001**
Malnourished	2 (0.5)	2 (1.5)	0 (0.0)	0 (0.0)	
At risk	71 (17.8)	42 (30.9)	14 (15.9)	15 (8.6)	
Normal	325 (81.7)	92 (67.6)	74 (84.1)	159 (91.4)	
IC total	5.05 ± 1.74	3.05 ± 1.03	5.00 ± 0.00	6.63 ± 0.68	**<0.001**
**IC domain decline**					
Locomotion	299 (75.1)	125 (93.4)	73 (83.0)	99 (56.9)	**<0.001**
Vitality	161 (40.5)	93 (68.4)	31 (35.2)	37 (21.3)	**<0.001**
Cognition	212 (53.2)	111 (81.6)	52 (59.1)	49 (28.2)	**<0.001**
Psychological	166 (41.7)	111 (81.6)	37 (42.0)	18 (10.3)	**<0.001**
**Physical performance**					
Max gait speed, m/s	0.95 ± 0.30	0.81 ± 0.28	0.93 ± 0.24	1.08 ± 0.28	**<0.001**
Slow gait speed	237 (59.5)	114 (83.8)	59 (67.0)	64 (36.8)	**<0.001**
Max handgrip strength, kg	21.67 ± 6.93	19.09 ± 6.35	22.38 ± 6.16	23.32 ± 7.17	**<0.001**
Low handgrip strength^1^	216 (54.3)	92 (67.6)	42 (47.7)	82 (47.1)	**0.002**
5x STS time (s)	13.17 ± 4.91	15.38 ± 6.34	13.75 ± 3.60	11.19 ± 3.18	**<0.001**
Poor 5x STS	205 (51.5)	94 (69.1)	54 (61.4)	57 (32.8)	**<0.001**
SPPB, total	9.66 ± 2.22	8.37 ± 2.41	9.51 ± 1.79	10.74 ± 1.62	**<0.001**
Poor SPPB	102 (25.6)	66 (48.5)	21 (23.9)	15 (8.6)	**<0.001**
**Body composition**					
ASMI, kg/m^2^	7.05 ± 2.36	6.46 ± 2.38	7.74 ± 3.32	7.19 ± 1.63	**<0.001**
Body cell mass, kg	25.81 ± 7.23	22.82 ± 6.31	26.47 ± 9.42	27.71 ± 6.05	**<0.001**
Whole body phase angle	5.48 ± 2.62	5.15 ± 2.48	6.08 ± 3.41	5.46 ± 2.27	0.146
Sarcopenia (AWGS)^2^	57 (14.3)	32 (23.5)	15 (17.0)	10 (5.7)	**0.007**

Values presented as *n* (%) or mean ± SD. BMI, body mass index; RAPA: rapid assessment of physical activity; MoCA, Montreal cognitive assessment; ADL, activities of daily living; IADL, instrumental activities of daily living; MNA, mini nutritional assessment; IC, intrinsic capacity; SPPB, short physical performance battery rest; STS, sit-to-stand; ASMI, appendicular skeletal muscle index; AWGS, Asian working group for sarcopenia.

^1^Adjusted for gender.

^2^Based on Asian Working Group for Sarcopenia (AWGS) 2019’s definition. Values shown in bold indicate statistical significance.

MoCA score was lowest in T1 (22.8 ± 5.2) followed by T2 (25.6 ± 4.0) and T3 (27.1 ± 2.6). The prevalence of malnutrition or at risk of malnutrition was 32.4% in T1, 15.9% in T2 and 8.6% in T3. Participants in T1 had the highest cognitive impairment rates (69.9% vs. 37.5% vs. 19.0%, respectively), depression rates (63.2% vs. 21.6% vs. 2.3%, respectively), at least 1 ADL (30.9% vs. 20.5% vs. 10.9%, respectively) and IADL impairments (41.9% vs. 21.6% vs. 19.0%, respectively).

The decline in locomotion domain was the most prevalent in T1 (93.4%) followed by T2 (83.0%) and T3 (56.9%). T3 compared to T2 and T1 participants had the fastest gait speed (1.1 ± 0.3 m/s vs. 0.9 ± 0.2 m/s vs. 0.8 ± 0.3 m/s), highest handgrip strength (23.2 ± 7.2 kg vs. 22.4 ± 6.2 kg vs. 19.1 ± 6.4 kg), shortest 5x sit-to-stand timing (11.2 ± 3.2 s vs. 13.8 ± 3.6 s vs. 15.4 ± 6.3 s) and highest SPPB performance (10.7 ± 1.6 vs. 9.5 ± 1.8 vs. 8.4 ± 2.4). T1 had the highest proportion of participants with slow gait speed (83.8% vs. 67.0% vs. 36.8%), low handgrip strength (67.6% vs. 47.7% vs. 47.1%), longer 5x STS timing (69.1% vs. 61.4% vs. 32.8%), and highest rates of poor SPPB performance (48.5% vs. 23.9% vs. 8.6%).

### Association of intrinsic capacity composite score with functional ability

For T1 compared with T3, IC composite score was significantly associated with at least 1 ADL impairment (aOR 3.36, 95% CI 1.78 to 6.32), IADL impairment (aOR 2.37, 95% CI 1.36 to 4.13), poor perceived health (aOR 0.96, 95% CI 0.95 to 0.98), at least 1 fall in the past year (aOR 1.63, 95% CI 1.05 to 2.84), cognitive impairment (aOR 8.21, 95% CI 4.69 to 14.39), and depression (aOR 101.82, 95% CI 33.62 to 308.37) (Table 2). For T2, only cognitive impairment (aOR 2.36, 95% CI 1.30 to 4.27), and depression (OR 12.97, 95% CI 4.20 to 40.00) had significant association.

**TABLE 2:** Association of intrinsic capacity composite score with functional ability and sarcopenia (Tertile 3 as reference).

	Tertile 1		Tertile 2	
	**Unadjusted**	**Adjusted^#^**	**Unadjusted**	**Adjusted^#^**
ADL impairment	**3.65 (2.00 to 6.64)**	**3.36 (1.78 to 6.32)**	**2.10 (1.04 to 4.24)**	1.91 (0.93 to 3.89)
IADL impairment	**3.08 (1.85 to 5.13)**	**2.37 (1.36 to 4.13)**	**1.18 (0.62 to 2.22)**	0.99 (0.51 to 1.91)
Perceived health	**0.97 (0.95 to 0.98)**	**0.96 (0.95 to 0.98)**	0.99 (0.97 to 1.01)	0.99 (0.97 to 1.01)
Cognitive impairment	**9.90 (5.85 to 16.77)**	**8.21 (4.69 to 14.39)**	**2.56 (1.44 to 4.55)**	**2.36 (1.30 to 4.27)**
Depression	**73.10 (25.56 to 209.09)**	**101.82 (33.62 to 308.37)**	**11.70 (3.84 to 35.65)**	**12.97 (4.20 to 40.00)**
Falls ≥ 1	**1.77 (1.07 to 2.93)**	**1.63 (1.05 to 2.84)**	0.89 (0.48 to 1.67)	0.88 (0.47 to 1.67)
ASMI	**0.73 (0.60 to 0.88)**	**0.78 (0.62 to 0.96)**	1.01 (0.92 to 1.11)	0.99 (0.89 to 1.12)
Body cell mass	**0.88 (0.83 to 0.92)**	**0.89 (0.84 to 0.95)**	**0.94 (0.90 to 0.99)**	**0.91 (0.85 to 0.97)**
Sarcopenia	**3.24 (1.50 to 6.97)**	**2.40 (1.06 to 5.45)**	**2.73 (1.14 to 6.53)**	**2.38 (1.07 to 5.89)**

Reference group: Tertile 3; Values presented as Odds Ratio (95% Confidence Interval).

Bold indicates significance (*p* < 0.05).

As defined by Asian Working Group for Sarcopenia 2019.

ASMI, appendicular skeletal muscle index.

^#^Adjusted for age, gender, ethnicity, education years and physical activity.

### Intrinsic capacity and sarcopenia

T1 participants had the lowest ASMI (6.5 ± 2.4 kg/m^2^ vs. 7.7 ± 3.3 kg/m^2^ vs. 7.2 ± 1.6 kg/m^2^), BCM (22.8 ± 6.3 kg vs. 26.5 ± 9.4 kg vs. 27.7 ± 6.1 kg) and highest prevalence of sarcopenia (23.5% vs. 17.0% vs. 5.7%). T1 was significantly associated with ASMI (aOR 0.78, 95% CI 0.62 to 0.96), BCM (aOR 0.85, 95% CI 0.79 to 0.91), and sarcopenia (aOR 2.40, 95% CI 1.60 to 5.45). T2 was significantly associated with BCM (aOR 0.91, 95% CI 0.85 to 0.97), and sarcopenia (aOR 2.38, 95% CI 1.07 to 5.89) (Table 2).

### Association of intrinsic capacity composite score with systemic inflammatory biomarkers

Serum GDF-15 was significantly elevated in T1 participants (Table 3). T1 had significant associations with GDF-15 (β 263.14, 95% CI 105.36 to 541.64), IL-10 (β−0.73, 95% CI −1.24 to −0.21), and IL-10 to TNF-α ratio (β−107.58, 95% CI −182.08 to −33.07). T2 also had significant associations with IL-10 (β−0.47, 95% CI −0.98 to −0.03) and IL-10 to TNF-α ratio (β−95.74, 95% CI −166.56 to −24.91) (Table 4).

**TABLE 3:** Baseline plasma biomarker levels.

	Overall *n* = 107	Tertile 1 *n* = 39 (36.4%)	Tertile 2 *n* = 24 (22.4%)	Tertile 3 *n* = 44 (41.1%)	*P*-value
GDF-15 (pg/mL)	876.10 (679.40)	1183.95 (1112.60)^a^	1051.40 (721.20)	692.80 (476.40)^a^	**0.030**
IL-6 (pg/mL)	2.70 (1.40)	2.85 (1.90)	2.80 (1.40)	2.70 (1.20)	0.883
IL-10 (ng/mL)	2.39 (1.32)	2.42 (1.33)	2.05 (1.42)	2.54 (1.44)	0.265
TNF-α (pg/mL)	7.50 (3.60)	8.40 (2.90)	7.80 (4.20)	6.90 (3.00)	0.116
IL-10 / TNF-α	311.48 (181.11)	276.50 (196.08)	291.15 (177.72)	346.47 (186.08)	0.088

Values presented as median (interquartile range).

*^a^*Values with common superscript alphabet are significantly different.

GDF-15: Growth Differentiation Factor-15. Bold indicates significance (*p* < 0.05). Data available for 107 participants.

**TABLE 4:** Univariate and multiple adjusted quantile regression for plasma biomarkers (Tertile 3 as reference).

	Tertile 1		Tertile 2	
	**Unadjusted**	**Adjusted^#^**	**Unadjusted**	**Adjusted^#^**
GDF-15	**480.00 (214.47 to 745.53)**	**263.14 (105.36 to 541.64)**	338.80 (−57.05 to 620.55)	170.89 (−93.84 to 435.62)
IL-6	0.10 (−0.48 to 0.68)	0.04 (−0.39 to 0.47)	0.10 (−0.52 to 0.72)	0.02 (−0.39 to 0.43)
IL-10	−0.08 (−0.64 to 0.48)	−**0.73 (**−**1.24 to**−**0.21)**	−0.49 (−1.09 to 0.11)	−**0.47 (**−**0.98 to**−**0.03)**
TNF-α	1.60 (−0.75 to 3.13)	0.83 (−0.38 to 2.03)	1.80 (−0.18 to 3.42)	0.61 (−0.54 to 1.75)
IL-10 / TNF-α	−67.16 (−154.55 to 20.23)	−**107.58 (**−**182.08 to**−**33.07)**	−45.08 (−137.81 to 47.65)	−**95.74 (**−**166.56 to**−**24.91)**

Reference group: Tertile 3.

Values presented as β (95% Confidence Interval).

Bold indicates significance (*p* < 0.05).

GDF-15: Growth Differentiation Factor-15.

^#^Adjusted for age, gender, ethnicity, education years, polypharmacy, physical activity, falls and IADL impairment.

## Discussion

This study represents one of the first few investigations into the prevalence of IC decline and its association with functional ability, sarcopenia, and systemic inflammation in pre-frail older adults. Participants in the highest tertile were significantly younger, better educated, and had higher cognitive scores. They also exhibited lower rates of depression, functional impairment, and malnutrition. Higher IC composite scores have shown to be associated with reduced frailty and disability progression (40). Our findings revealed that 95% of pre-frail older adults experienced at least one IC domain decline, slightly surpassing the 89.9% prevalence observed in participants with sarcopenia and the 83.3% prevalence in pre-frail older adults from China at baseline (14, 16). Notably, the proportion of pre-frail older adults with declines in individual IC domains was nearly triple that of the original ICOPE Pilot validation study in China: 75.1% compared to 25.3% in locomotion, 40.5% compared to 16.2% in vitality, 53.2% compared to 46.8% in cognition, and 41.7% compared to 12.0% in the psychological domain (41). This could be attributed to different population group where the participants from the ICOPE Pilot validation study were from hospitalized cohort, had a lower mean age, and were evaluated using simplified measurement tools such as 5x-STS for mobility and 3-item recall and orientation for cognitive decline. Given the significant heterogeneity in measurement of individual domains, there has been a recent collection of publication in collaboration with the WHO on standardizing healthy aging assessments (42).

Locomotor impairment was prevalent across all tertiles, with 93% of participants in T1 and 56.9% in T3 exhibited low gait speed or 5x-STS. In a 2-year longitudinal study, new impairment in locomotion and vitality were associated with progression from non-frail to frail status (16). Another study reported that impaired locomotion and vitality at baseline were associated with “kept frail” or “worsened frailty status” (6). While the domains are separate entities and interventions may be domain specific, there are significant interactions between domains and decline in one can impact decline in another with cumulative impact on functional ability (8, 43). Our study demonstrated this interplay: participants in T1 were three times more likely to have cognitive impairment, eight times more likely to have a decline in psychological domain and four times more likely to be sarcopenic.

Functional ability is one of the key measurement of the success of the Decade of Healthy Aging action plan which was declared by the United Nation (1, 4). Lower composite scores were significantly associated with functional and cognitive impairment, depression, poor perceived health and falls. Stolz et al. (12) showed significant heterogeneity in IC decline over 21 years where 1 point decline was associated with 7% increase in risk of ADL disability, 6% increase in nursing home admission and 5% increased risk of mortality (12). Studies have reported significant variability in domains which may predict adverse outcomes in different populations. In the Multidomain Alzheimer Preventive Trial (MAPT) cohort, mobility decline, depression and visual impairment were associated with higher incidence of frailty over 5 years, and each additional decline in IC was associated with higher incidence frailty by 47%, IADL decline by 27%, and ADL decline by 23% (8). Yu et al. (44) reported that cognitive decline, limited mobility, visual impairment and depression predicted incident disability whereas cognitive decline and limited mobility predicted emergency department visits amongst Chinese community dwelling older adults over 1 year (44). On the other hand, low nutrition scores and low balance performance predicted 3-year mortality and falls in nursing home residents in Belgian (45).

Lower tertiles of IC composite score were significantly associated with sarcopenia and muscle mass indices such as body cell mass and appendicular skeletal muscle mass. Sarcopenia has shown to be associated with individual IC domains such as hearing, depression, dementia, functional mobility, and vision (11, 46, 47). Notably, only one study has explored the association between IC composite score and sarcopenia but in hospitalized older adults whereas our study population were community dwelling older adults (14).

Despite the growing interest in IC, limited research has explored its associations with systemic inflammation. In this study, we examine the link between IC composite scores and specific biomarkers. Lowest tertile of IC composite score demonstrated significant associations with high GDF-15, low IL-10, and IL-10 to TNF-α ratio. Secondary analysis from the MAPT study with longitudinal follow up over 60 months revealed that rapid decline in IC trajectory correlated with TNFR-1, GDF-15, and monocyte chemoattractant protein-1 (21). Our results are in keeping with other studies which showed significant relationship of GDF-15 with sarcopenia, frailty, gait speed and poor physical function in older adults (48). A prior systematic review did not show association of IL-10 with frailty possibly due to limited data as only nine studies were included on IL-10 in and frailty (20). Interestingly, IL-10 knockout mice exhibited increased expression of serum IL-6, and faster muscle strength decline (24). TNF-α/IL-10 ratio serves as a surrogate for immune homeostasis which measures ratio of pro- and anti-inflammatory cytokines. Prior studies have shown that high TNF-α/IL-10 ratio have been associated with frailty, motoric cognitive risk syndrome, severity of burn injury and susceptibility to infections in burn patients (25, 49). TNF-α and IL-6 did not significantly differ between groups, possibly due to enrolled participants being pre-frail (20). In addition, prior studies reported significant association of frailty and IC with TNFR-1 which is widely expressed and implicated in cell death, and inflammation (50).

Our study further strengthens the concept of multidimensional nature of IC in pre-frail older adults and the impact of composite score on functional ability and systemic inflammation. However, there are several limitations which warrant mention. First and foremost, there is no gold standard for diagnosis of sarcopenia. Muscle mass measurement was made using the multi-frequency bioelectrical impedance analyzer which includes intramuscular fat, fibrotic and connective tissue. The D3 Creatine dilution method better predicts muscle mass and associated with functional status (51). Second, we lack information on the surrounding environment which can impact IC, participation restriction, hearing, and vision. These factors may impact other domains and overall scores. However, Liu et al reported that newly impaired locomotion and vitality were significantly associated with frailty progression (16). Both vision and hearing impairment have been shown to be associated with sarcopenia. Third, the cross-sectional nature of the study limits causal association. Fourth, our study population were pre-frail limiting generalizability to the broader population. Fifth, the data on chronic disease, falls, function, and medications were based on self-report and maybe subject to recall bias. Sixth, as our study participants were ambulant and able to follow instructions, the association of IC with functional ability maybe under-reported. Seventh, sarcopenia diagnosis was made based on the Asian Working Group for Sarcopenia 2019 Consensus which recommended using the 6-meter walk; we used the 4-meter walk with a 3-meter of acceleration and deceleration path. It is known that multiple factors can affect gait speed such as distance, flooring surface, automatic vs. manual timing, clinical condition, endurance and starting test procedures. A systematic review reported a non-clinically significance median difference of 0.04 m/s between longer and shorter distance (52). Lastly, while SCD is recognized as a cognitive testing instrument, reporting of SCD may vary between different population and ethnic groups (40, 53).

It is becoming increasingly evident that both composite and individual domain scores are important in planning personalized interventions and measuring outcomes. Functional ability is determined by the inter-relationship between different IC domains where composite scores may be valuable in measuring impact of multidomain interventions or the impact of the Decade of Healthy Ageing action plans on quality of life, physical function, cognition, and mental health. Emerging studies show that baseline IC and inflammation better predict the onset of disability (21). In clinical practice, IC composite scores may be able to stratify surgical risk, guide medical treatment and rehabilitation strategies. However there are limited scientific publications in this field (42). Many countries are implementing public health program like the INSPIRE integrated care for older people (ICOPE)-CARE programme in Occitanic for screening of IC with personalized management (54). However, there are significant gaps which needs to be addressed such as validated screening tools for individual domains before routine screening at population level can be implemented. One such example is vitality where studies have used measures such as HGS, weight loss, fatigue, and nutrition. In addition, the association with functional ability and biomarkers need to be validated in longitudinal studies. Nonetheless, IC composite scores could potentially serve as a measure of biological aging.

## Conclusion

The IC domains composite score was significantly associated with functional ability, perceived health, sarcopenia, and systemic inflammation biomarkers such GDF-15, IL-10, and IL-10/TNF-α ratio in pre-frail older adults. Future prospective longitudinal population studies are needed to validate association of sarcopenia and functional ability with IC composite scores.

## Data availability statement

The raw data supporting the conclusions of this article will be made available by the authors, without undue reservation.

## Ethics statement

The studies involving humans were approved by the National Healthcare Group Domain Specific Review Board (Reference 2018/01183 and 2019/00017). The studies were conducted in accordance with the local legislation and institutional requirements. The participants provided their written informed consent to participate in this study.

## Author contributions

RM: Conceptualization, Data curation, Formal Analysis, Funding acquisition, Investigation, Methodology, Project administration, Resources, Software, Supervision, Validation, Visualization, Writing – original draft, Writing – review and editing. YC: Data curation, Formal Analysis, Writing – original draft, Writing – review and editing. DA: Formal Analysis, Writing – original draft, Writing – review and editing. BV: Supervision, Visualization, Writing – original draft, Writing – review and editing.
