# Supplementary material for: Association of intrinsic capacity with functional ability, sarcopenia and systemic inflammation in pre-frail older adults
Source: Front Med (Lausanne). 2024 Mar 6;11:1374197. doi: 10.3389/fmed.2024.1374197 (PMC10953915; doi:10.3389/fmed.2024.1374197)
Supplement: Supplementary file 1 [file Data_Sheet_1.docx]

Supplementary material

**Supplementary Table 1.** Intrinsic capacity domains and scoring

| **Domain** | **Component** | **Intrinsic Capacity Scoring (Total 8)** | | |
| --- | --- | --- | --- | --- |
|  |  | **0** | **1** | **2** |
| Locomotion | Gait Speed and 5x Sit-to-Stand (STS) Timing | Gait Speed <1m/s and 5x STS <12s | Either Gait Speed <1m/s or 5x STS <12s | Neither Gait Speed <1m/s nor 5x STS <12s |
| Vitality | Nutritional Status and ASMI | Malnourished / at risk of malnutrition and  Low ASMI | Either malnourished/at risk of malnutrition or Low ASMI | Neither malnourished/at risk of malnutrition nor Low ASMI |
| Cognition | MoCA and SCD | MoCA <26 and SCD | Either MoCA <26 or SCD | Neither MoCA <26 nor SCD |
| Psychological | Depression and EQ5D question on anxiety/depression | Depression and EQ5D anxiety/depression >1 | No depression and EQ5D anxiety/depression >1 | No depression and EQ5D anxiety/depression ≤ 1 |

**Supplementary Table 2.** Decline in Intrinsic Capacity (by number of domains)

| Number of domains decline | % |
| --- | --- |
| 1 | 95.0 |
| 2 | 68.6 |
| 3 | 34.5 |
| 4 | 12.6 |
